# Cell–Cell Communication in Embryonic Kidney Development: Insights from Single-Cell and Spatial Transcriptomics

**DOI:** 10.3390/jdb14040043

**Published:** 2026-10-02

**Authors:** Leping Wang, Xuejia Zheng, Feng Zhu, Liu Xiang, Chengcheng Liu, Enqi Feng, Yuxian Liu, Yong Dai

**Affiliations:** 1 School of Mathematics and Big Data, Anhui University of Science and Technology, Huainan 232001, China; 17330699973@163.com (L.W.);; 2 The First Hospital, Anhui University of Science and Technology, Huainan 232001, China; 3 School of Computer Science and Engineering, Anhui University of Science and Technology, Huainan 232001, China; 4 School of Artificial Intelligence, Anhui University of Science and Technology, Hefei 231131, China; 5 State Key Laboratory of Digital Intelligent Technology for Unmanned Coal Mining, Anhui University of Science and Technology, Huainan 232001, China; 6 School of Medicine, Anhui University of Science and Technology, Huainan 232001, China; 7 Anhui Provincial Academician Workstation of Anhui University of Science and Technology for Autoimmune Disease Diagnostic Technology, Huainan 232001, China

**Keywords:** dynamic developmental niche, embryonic kidney development, ligand–receptor interaction, single-cell RNA sequencing, spatial transcriptomics

## Abstract

Embryonic kidney morphogenesis depends on the coordinated communication among ureteric epithelium, nephron progenitors, stroma, endothelium and differentiating nephrons. Classical studies established central roles for glial cell line-derived neurotrophic factor (GDNF) signaling through the RET proto-oncogene receptor tyrosine kinase (RET), WNT-family signaling, fibroblast growth factor (FGF), bone morphogenetic protein (BMP), Notch, Hedgehog, vascular endothelial growth factor (VEGF) and platelet-derived growth factor (PDGF) pathways, but pathway-centered models do not fully explain why similar signals produce different outcomes across cell states, anatomical locations and developmental stages. Single-cell RNA sequencing (scRNA-seq) and spatial transcriptomics have refined these models by resolving heterogeneous progenitor, stromal and vascular states, reassigning several signal sources and constraining predicted interactions to defined tissue relationships. Here, we examine how these findings change the interpretation of ureteric bud (UB) branching, nephron progenitor transitions, stromal patterning and vascular integration. We use the dynamic developmental niche as an evidence-organizing framework, rather than as a new biological entity, to distinguish established mechanisms from relationships supported mainly by transcriptomic, spatial or computational evidence. Vascular, immune and mechanical interactions are evaluated according to their model-specific support, and we outline experiments required to connect predicted communication with morphogenetic outcomes and congenital anomalies of the kidney and urinary tract (CAKUT).

## 1. Introduction

Kidney morphogenesis depends on sustained and precisely coordinated communication among the ureteric bud (UB) epithelium, metanephric mesenchyme, nephron progenitor cells (NPCs), stroma, and vasculature [1]. Canonical pathways including GDNF–RET, WNT, FGF, BMP, Notch, and Hedgehog jointly regulate UB branching, nephron progenitor maintenance, mesenchymal-to-epithelial transition, and nephron segmentation [1,2,3]. These studies established the basic molecular framework of kidney development, but pathway-centered models alone cannot readily explain why the same signal produces different outcomes across cellular states, tissue locations, and developmental stages.

Single-cell RNA sequencing (scRNA-seq) and spatial transcriptomics have provided new evidence with which to address this problem [4]. Single-cell studies have resolved continuous states within nephron progenitor, stromal, and endothelial populations previously regarded as homogeneous, and have reassigned several signals to more precisely defined sender and receiver cells [5,6]. Spatial approaches place these transcriptional states within specific structures, including UB tips, the nephrogenic zone, stromal boundaries, and vascular territories, thereby distinguishing transcriptionally possible interactions from anatomically plausible communication [7]. Beyond expanding the cellular atlas of the developing kidney, these advances have prompted classical pathways to be reinterpreted in the context of cell state and tissue geometry. Existing reviews have primarily examined developmental signaling pathways, lineage relationships, species conservation or organoid differentiation [8,9]. Here, we instead evaluate how cell–cell communication is assigned to defined sender and receiver populations, how these assignments are constrained by developmental stage and tissue position, and what evidence is required to distinguish an inferred interaction from a functional morphogenetic mechanism.

To connect cell atlases with morphogenetic mechanisms, we use the dynamic developmental niche as an operational framework for evaluating local developmental communication. Its contribution is analytical rather than ontological: it does not rename the developmental microenvironment or propose an additional biological structure. Instead, it separates a pathway-level claim into defined sender identity, signal availability, receiver competence, developmental timing, anatomical range and measurable response. Applied across studies, these criteria reveal three recurring limitations in the current literature: uncertain cellular sources of established developmental signals, incompletely defined receiver competence and insufficient evidence connecting predicted interactions to morphogenetic outcomes. The review therefore focuses on the composite progenitor–stromal GDNF source, state-dependent WNT responsiveness and the restricted evidence linking stromal NTN1 to renal vascular organization.

The review examines how these principles apply to UB branching, nephron induction, stromal and vascular development, model-system boundaries and congenital anomalies of the kidney and urinary tract (CAKUT).

## 2. Literature Search and Selection Strategy

This narrative review was compiled through literature searches of PubMed, Web of Science and Google Scholar. The search terms included “kidney development”, “embryonic kidney”, “metanephric kidney”, “ureteric bud”, “nephron progenitor”, “cap mesenchyme (CM)”, “scRNA-seq”, “spatial transcriptomics”, “cell–cell communication”, “ligand–receptor interaction”, “CellChat”, “CellPhoneDB”, “NicheNet”, “kidney organoid” and “CAKUT”. We prioritized studies that provided mechanistic insights into kidney morphogenesis, single-cell or spatial transcriptomic analyses of fetal or embryonic kidney development, computational inference of intercellular communication, and experimental validation using genetic perturbation, imaging, lineage tracing or organoid models. Seminal studies on kidney developmental signaling were also included when they provided essential background for interpreting communication modules. Because this article is a narrative and conceptual review, no meta-analysis or formal quality scoring was performed.

## 3. Modes and Inference of Intercellular Communication in Kidney Development

### 3.1. Autocrine, Paracrine, Endocrine and Contact-Dependent Signaling in the Developing Kidney

Local kidney morphogenesis is coordinated mainly through paracrine, autocrine and contact-dependent signaling. Mesenchymal GDNF acts on RET–GFRα1-positive UB tip cells, whereas UB-derived WNT9B promotes nephron induction in adjacent mesenchyme [2]. Notch-mediated contact contributes to nephron segmentation and cell-fate specification [3]. Systemic and maternal influences may modify the broader developmental environment but cannot usually be assigned to local sender–receiver relationships using embryonic-kidney transcriptomic data [10]. Table 1 summarizes the principal local communication axes and their evidence.

### 3.2. Inferring Communication from Single-Cell and Spatial Transcriptomic Data

Using scRNA-seq, candidate ligand production and receptor expression can be assigned to defined populations within the developing kidney. This approach helps distinguish potential sender and receiver cells among the UB epithelium, nephron progenitors, stroma, endothelium and differentiating nephron epithelia. Methods such as CellPhoneDB, CellChat and NicheNet organize these expression patterns into ligand–receptor pairs, pathway-level networks or ligand-associated transcriptional responses [20,21,22]. Their main value is to generate testable relationships with a specified cellular source and developmental context, rather than to produce an extensive catalog of possible interactions [23].

Spatial transcriptomics adds anatomical information to these candidates. A predicted interaction becomes more plausible when the relevant cells occupy compatible positions at the same developmental stage. The required spatial relationship depends on the mode of communication: contact-dependent signaling requires direct adjacency, whereas secreted or matrix-associated factors may act across a local tissue domain. Spatial information therefore narrows the set of candidate relationships and helps distinguish interactions associated with UB tips, the nephrogenic zone, stromal boundaries or developing vessels [7]. It does not, by itself, establish that signaling has occurred. Communication inference becomes useful when it addresses a defined developmental question. Table 1 therefore records the model, stage, sender, receiver, evidence type and morphogenetic outcome for each major axis.

Throughout this review, we distinguish four types of claims. A technology-enabled observation is directly resolved by single-cell or spatial analysis but does not by itself establish function. An experimentally supported mechanism links a defined developmental response to targeted perturbation, rescue or comparable functional evidence. A literature reinterpretation integrates findings from several studies into a more specific explanation that has not yet been tested as a complete mechanism. A prospective hypothesis extends the available evidence into a testable relationship for future investigation. These categories define the origin and current status of a claim, whereas evidence levels indicate the strength of support for the specific relationship being evaluated.

## 4. How Single-Cell and Spatial Technologies Have Revised Models of Kidney Development

Using these distinctions, we next examine which aspects of kidney-development models have been revised by single-cell and spatial technologies. Classical genetic studies established the essential roles of GDNF–RET, WNT, FGF, BMP, Notch and hedgehog signaling in UB branching, nephron progenitor maintenance and nephron formation [1]. Single-cell and spatial omics have extended this framework by revising assumptions about signal source, receiver-cell identity and developmental timing [4]. In the most informative cases, single-cell data reveal previously unrecognized cellular states or signaling sources, spatial analyses define their anatomical relationships, and genetic or functional studies determine whether these observations alter the interpretation of morphogenesis. The following sections therefore distinguish three types of advances enabled by high-dimensional datasets: revised cellular source assignment, resolution of state-dependent receiver populations and identification of communication relationships that now require direct functional testing.

### 4.1. Nephron Progenitors Are Not a Homogeneous, Synchronously Responding Population

Early models generally treated the SIX2-positive cap mesenchyme as a functionally uniform progenitor pool that shifts from self-renewal to nephron formation following WNT induction. Human fetal kidney scRNA-seq instead resolves proliferative, self-renewing, primed and pretubular aggregate-transition states within the nephron progenitor cell (NPC) compartment [6,24]. These data define transcriptional composition and developmental ordering; they do not show that the inferred states are stable lineages or that a particular signal causes movement between them.

Spatial analyses place this transcriptional continuum within the sequence of nephron morphogenesis. In the human fetal kidney, the distributions of CDH1 and JAG1 indicate that epithelial features emerge progressively while mesenchymal cells continue to enter the forming nephron. Three-dimensional mapping further resolves precursor domains with proximal, distal, podocyte or parietal epithelial bias [25]. These observations establish where and when molecular states occur. They do not establish the signals that generate those states.

This organization changes how WNT responses should be interpreted. WNT9B remains a central inductive signal, but a ligand concentration outside the cell is not equivalent to intracellular pathway activity. Experiments using CHIR99021 vary β-catenin pathway activation downstream of the receptor and show dose-dependent responses in cultured mouse NPCs [26]; they do not measure an in vivo WNT9B concentration threshold. Receiver competence may vary with NPC state, receptor and co-receptor abundance, developmental stage, FGF/BMP context, cell-cycle position and chromatin state [27]. No study has established a fixed WNT threshold shared by all NPCs.

### 4.2. Single-Cell Analysis Has Reassigned Several Signal Sources

The spatial evidence supports a composite sender domain rather than a single-source model [24]. NPCs remain an important source of GDNF, but adjacent stroma may also contribute to the distribution of the signal received by the UB tip. This distribution is likely to depend on the relative contribution of each cellular source, local tissue geometry and extracellular-matrix binding, and cannot be inferred from whole-kidney Gdnf expression alone. Stromal expression is supported by cross-platform and spatial evidence. The respective contributions of *Six2-* and *Foxd1*-lineage cells to budding, elongation and bifurcation, however, have not been quantified through temporally controlled deletion on a shared experimental background. The evidence therefore supports stromal participation in GDNF production, but not yet a stage-specific allocation of function between the two sources.

The broader reassessment of the renal stroma follows a similar progression [28]. Classical descriptions often treated FOXD1-positive stroma as a relatively uniform support compartment. Single-cell atlases distinguish nephrogenic-zone, cortical and medullary stromal states in the mouse, as well as outer cortical, nephrogenic-zone-adjacent, inner cortical and medullary-biased populations in the human fetal kidney [29]. Their expression of extracellular-matrix components, chemokines, axon-guidance molecules and growth factors differs with anatomical position, suggesting regionally distinct signaling capacities [5,6]. Because stromal populations are divided and named differently across datasets, their correspondence should be assessed using spatial location and inferred function rather than cluster labels alone.

### 4.3. Communication Axes with Different Levels of Functional Support

Functional experiments strengthened this cellular and spatial assignment. Stromal deletion of *Ntn1* disrupted renal arterial branching, reduced vascular smooth-muscle coverage and impaired vessel maturation rather than simply eliminating endothelial cells [19]. These vascular abnormalities were accompanied by abnormal kidney development. The evidence therefore supports a role for stromal NTN1 in organizing renal vascular pattern and indicates that the observed defects cannot be explained by VEGFA abundance alone. The relationship between the vascular phenotype and the accompanying nephron abnormalities nevertheless remains more difficult to resolve.

The Hedgehog–CXCL12/WNT5A relationship reached mechanistic support through a different route. Sustained Hedgehog activation in Foxd1-positive stroma produces stromal expansion and reduces nephron number. scRNA-seq of the altered stromal compartment identified *Cxcl12* and *Wnt5a* as candidate downstream factors. Genetic reduction of either factor in the same experimental context subsequently improved nephron formation [16]. Hedgehog activation in human kidney organoids produced directionally concordant stromal expansion and nephron reduction [16].

These observations move the Hedgehog–CXCL12/WNT5A relationship beyond ligand–receptor co-expression because the predicted mechanism is supported by cell-type-specific perturbation and genetic rescue. They support a causal contribution of abnormal stromal signaling to impaired nephron formation. Its physiological dose range and principal receiver populations during normal human kidney development, however, remain unresolved.

These examples represent distinct evidence levels, ranging from spatial source assignment to lineage-specific perturbation and rescue, as summarized in Table 1.

### 4.4. Spatial Atlases Refine Interpretations of Cell Identity and Proximity

Single-cell studies have identified substantial transcriptional similarity between the distal nephron and ureteric epithelium. Markers such as GATA3, E-cadherin, PAX2 and HOXB7-related genes have been used to assign ureteric epithelial identity, although whole-transcriptome comparisons show that several are also expressed in the distal nephron and connecting segment. Classification based on a single marker can consequently misidentify distal nephron cells as UB or collecting duct epithelium [30,31].

This distinction is particularly important in kidney organoids. The distal nephron epithelium can exhibit plasticity in vitro and acquire selected RET-positive, ureteric-tip-like features under particular culture conditions. Such changes demonstrate phenotypic plasticity but do not establish a ureteric lineage origin in vivo [31]. Cell identity therefore requires support from broader transcriptional programs, anatomical position and developmental trajectory rather than isolated marker expression.

Spatial data provide an additional level of discrimination by showing that transcriptionally similar cells can occupy different anatomical relationships. During formation of the human S-shaped body, tissue curvature brings presumptive distal cells close to the glomerular region and positions several domains that later participate in local physiological communication [25,32]. Vascular endothelium subsequently enters the glomerular cleft and establishes the podocyte–endothelial interface. This proximity is generated by morphogenesis rather than represented by a static cell map, and it cannot be reconstructed reliably from transcriptional similarity after tissue dissociation.

## 5. Dynamic Developmental Niches with Defined Boundaries: Local Communication and Process-Specific Transitions

Building on the revised cellular assignments and evidence boundaries described in Section 4, this section examines how these observations can be organized into process-specific communication models without transferring pathway-level evidence to untested niche-level claims.

### 5.1. Operational Boundaries and Interpretive Value of a Dynamic Developmental Niche

**Figure 1:**
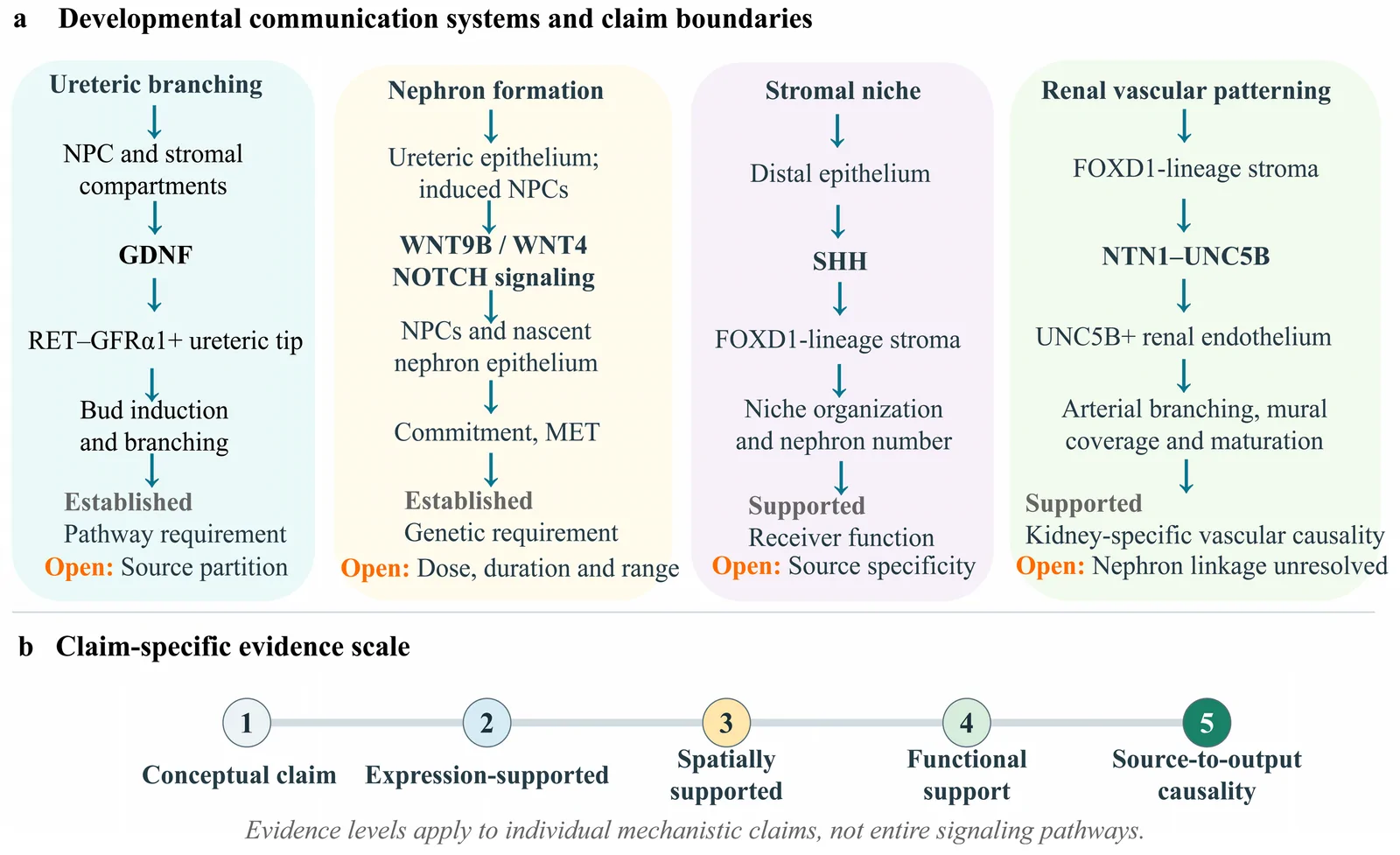
Claim-specific evidence map of developmental communication systems in the embryonic kidney. (**a**) Four representative developmental communication systems are presented as separate modules linking a sender population, signaling axis and receiver state to a measurable morphogenetic output, together with the claim-specific evidence boundary. (**b**) The evidence scale ranges from conceptual claims to source-to-output causality. Evidence levels apply to individual mechanistic claims rather than to entire signaling pathways. The illustrated systems summarize selected developmental relationships and should not be interpreted as complete or uniformly established causal chains.

A dynamic developmental niche is defined here as a local communication system that operates within a specified developmental interval and anatomical range. It requires an identifiable sender, an available signal, a competent receiver and a measurable cellular or morphogenetic response. Proximity and temporal overlap create an opportunity for interaction, but they do not demonstrate signaling. A transcriptional cluster, spatial domain or ligand–receptor co-expression pattern is not considered a functional niche by itself. These claim-specific evidence boundaries are summarized in Figure 1.

The framework differs from a general description of the developmental microenvironment because it evaluates each component as a separate evidential claim. A pathway may be required for development even when its cellular source, effective range or stage-specific receiver response remains uncertain. Likewise, co-expression and spatial proximity establish an opportunity for communication but do not demonstrate pathway activation or morphogenetic function. The relevant criteria therefore include sender identity, signal availability, receiver competence, developmental timing, anatomical range and measurable response. Evaluating these components separately identifies which part of a proposed communication chain is supported and which part requires further testing. These criteria define interpretive boundaries rather than a separate biological mechanism.

The communication system may be modified by the extracellular matrix (ECM), tissue mechanics, oxygen availability, nutrient supply or metabolic state. These factors belong in a specific niche model only when they can be linked to signal availability, receiver competence or a defined developmental output. Vascular and immune cells should likewise be assigned as specific senders, receivers or functional modifiers rather than treated as generic environmental components. Cell-autonomous programs may influence receiver competence without constituting intercellular communication. The following sections apply these boundaries to individual developmental processes and retain relationships without adequate functional support as candidate interactions or testable hypotheses.

### 5.2. The UB Tip Niche: Transitions from Budding to Branching

Section 4.2 identifies a composite nephron progenitor–stromal GDNF sender domain as a revised cellular assignment [33]. The remaining question is whether this assignment has a stage-specific morphogenetic function during ureteric bud branching. The UB tip niche can therefore be evaluated as a response-boundary problem, in which local GDNF availability, RET–GFRα1 competence, negative feedback and tissue geometry together determine the probability of budding or bifurcation.

As tip cells progress through branch initiation, elongation and bifurcation, their morphology and signaling state change together [34,35]. Branch curvature, cell rearrangement and stromal boundaries may influence local tension and ligand accessibility, whereas receptor-mediated negative feedback limits excessive or ectopic budding. The UB tip niche can therefore be viewed as a moving response interface. Its output is better assessed by tip number, extension rate, branch angle and bifurcation probability than by RET target-gene expression alone.

### 5.3. The Nephron Progenitor Niche: A Moving Boundary Among Self-Renewal, Priming, and Induction

Section 4.1 shows that nephron progenitors occupy a continuum of self-renewing, primed and transition states [36]. Within this continuum, the central unresolved issue is whether matched progenitor states exhibit distinct responses to defined WNT input intensity and duration. UB-derived FGF and WNT signals, together with stromal BMP signals and other local regulators, provide the developmental context in which this question should be tested.

The quantitative terms describe different variables. Ligand dose is the extracellular amount of WNT available to its receptors; exposure duration is the time over which that input is present; and cumulative exposure is its time-integrated history. Pathway activity is the receiver response, measurable through nuclear or reporter-based β-catenin/TCF activity, and an operational response boundary is the input at which a specified outcome changes under defined conditions. Receiver competence includes receptor abundance and the cell state that permits or restricts that response. These variables cannot be used interchangeably.

The present framework does not provide a mathematical model, a predictive equation or a universal response threshold. It identifies the variables and claim-specific measurements required for future quantitative modeling. Current studies differ in experimental system, developmental stage, pathway input and outcome definition; their numerical estimates therefore cannot yet be combined into a common input–response function.

A test of state-dependent WNT responsiveness should record the initial SIX2/CITED1 state and measure early β-catenin activity, Wnt4 induction, progenitor exit, pretubular aggregate formation and mesenchymal-to-epithelial transition. Developmental stage, FGF/BMP background, receptor abundance, cell cycle, chromatin state, viability and total growth should be controlled. The model is supported only if aligned NPC states retain reproducibly different input–duration response boundaries; equivalent responses after state alignment would weaken it.

Metabolic state may modify nephron progenitor competence without constituting an intercellular communication mechanism by itself. Oxygen availability, nutrient supply and intracellular metabolic activity could alter the capacity of a progenitor cell to respond to WNT, FGF or BMP inputs, but kidney-specific evidence linking a defined metabolic change to a particular signaling response and morphogenetic outcome remains limited. Expression of metabolic genes or enrichment of a metabolic pathway therefore cannot establish that metabolic input causes a change in progenitor fate. Such a claim would require controlled manipulation of oxygen, nutrient availability or a defined metabolic process, together with measurements of cell viability, progenitor state, pathway activation and nephron formation. Vascular paracrine signaling should also be distinguished experimentally from the effects of vascular delivery of oxygen and nutrients. Within the present framework, metabolism is therefore treated as a potential modifier of receiver competence rather than as an established developmental communication axis.

### 5.4. Stromal Communication and Modulation by the ECM and Tissue Mechanics

The renal stroma contains spatially distinct populations with different developmental functions [4]. Cortical stroma lies adjacent to the nephrogenic zone, whereas medullary and perivascular populations contribute to internal tissue organization and the developing vascular environment. These cells influence neighboring epithelia and nephron progenitors through secreted factors, signaling antagonists and extracellular-matrix components. Stromal boundaries can therefore restrict the location of signal-producing cells while also defining the physical environment through which cells migrate and tissues change shape [37].

The ECM is remodeled throughout kidney development and contributes to epithelial adhesion, polarity and force transmission [38]. Kidney-specific experiments support several parts of this relationship. Conditional manipulation of the NF2–Hippo–YAP/TAZ pathway in the mouse ureteric epithelium alters tip identity and branching [39], while UB deletion of integrin β1 causes a severe branching defect and impairs growth-factor-dependent signaling [40]. These genetic findings establish roles for Hippo signaling and cell–ECM adhesion in renal morphogenesis. They do not show that matrix stiffness controls RET responsiveness.

Evidence for focal adhesion kinase (FAK)–Src signaling is more limited in model depth. A hierarchical study using embryonic collecting-duct cell lines, three-dimensional tubulogenesis assays and mouse kidney explants found that FAK–Src inhibition reduced collecting-duct growth and branching [41]. This supports pathway involvement in a kidney-derived experimental setting, but does not identify the relevant mechanical input or demonstrate a stiffness-dependent change in GDNF–RET signaling. Direct links among matrix stiffness, tissue tension and YAP/TAZ activity are better established in other branching organs. Those studies provide mechanistic hypotheses for the kidney rather than kidney-specific confirmation.

Stromal abnormalities may alter communication through both tissue geometry and biochemical context. A weakened stromal boundary could change matrix organization, curvature or ligand distribution without reducing total GDNF expression. Conversely, altered GDNF–RET activity could modify epithelial growth and branch shape, thereby changing local tension and matrix loading. A niche transition can therefore be interpreted only when the temporal order of geometry, mechanical state, ligand localization, RET activity and branch behavior is defined in the same preparation. This approach distinguishes a potential initiating mechanical change from feedback generated by epithelial growth.

### 5.5. Vascular Integration: Paracrine Signaling, Tissue Supply, and Stromal Regulation

Renal vascularization and nephrogenesis overlap in time and space. Endothelial entry, vascular-network remodeling, arterial maturation and mural-cell recruitment occur while epithelial and nephron structures are still forming [42]. Single-cell data distinguish immature, arterial-like and venous-like endothelial states, but the coincident appearance of these populations and nephron maturation does not establish that endothelial signals initiate nephron differentiation [43]. A vascular model must therefore distinguish signals that precede vascular entry, endothelial paracrine activity and the tissue-level effects of oxygen and nutrient delivery after vessels have formed.

Among the vascular communication mechanisms considered here, stromal NTN1 signaling to endothelial UNC5B has the clearest kidney-specific causal support. The accompanying nephron abnormalities may reflect altered perfusion, stromal state, vascular signaling or a combination of these effects, and their causal order remains unresolved.

VEGFA–KDR and PDGF-B–PDGFRβ signaling also have functional support in renal vascular development. VEGFA contributes to the recruitment and maintenance of glomerular endothelium, whereas endothelial PDGF-B signaling supports the recruitment of PDGFRβ-positive mural and mesangial-lineage cells [17,18]. These mechanisms should not be generalized into a single model of vascular entry throughout the nephrogenic zone.

ANGPT1–TEK signaling is well characterized in vascular stabilization more broadly, but its stage-specific cellular sources and kidney-intrinsic functions during embryonic renal vascularization remain less clearly defined [44,45]. ANGPT2–TEK signaling is associated with context-dependent endothelial remodeling in other vascular systems, whereas its timing and functional requirement in the developing kidney remain incompletely established. Neither relationship should be described as a confirmed trigger of nephron formation.

Vascular maturation also changes local oxygenation, nutrient supply and waste clearance. These tissue-level effects must be separated from endothelial paracrine signaling before vascular control of nephron differentiation can be inferred. This distinction requires independent manipulation of perfusion and endothelial signaling, with measurements of local oxygenation, progenitor state and nephron formation.

### 5.6. Immune Cells During Development: Candidate Modulators of Local Developmental Processes

Single-cell and spatial analyses establish that immune cells are present in the human fetal kidney before nephrogenesis is complete. Macrophages and dendritic-cell populations are detected between 7 and 16 post-conception weeks, with lymphoid populations appearing later and showing stage-dependent regional distributions [46]. These observations define cellular presence, timing and anatomical association. They do not show that spatially adjacent immune and renal cells communicate or that immune cells regulate nephron formation.

The developmental origins of renal macrophages remain heterogeneous. Mouse lineage and fate-mapping studies support contributions from yolk-sac erythromyeloid progenitors and fetal-liver-derived cells, whereas human origins are inferred from developmental timing and transcriptional similarity rather than direct lineage tracing [47]. The source of a macrophage population should therefore be separated from its function within the kidney.

Kidney-specific experiments provide limited but direct evidence for selected macrophage functions. In mouse embryos, genetic macrophage ablation impaired clearance of misplaced NPCs, and antibody-mediated depletion in kidney explants reduced vascular anastomoses [48]. These findings support roles in progenitor-field organization and vascular connectivity under the tested conditions. They do not establish that macrophages directly control nephron induction or branching through CSF1–CSF1R, CX3CL1–CX3CR1 or another defined ligand–receptor axis. Expression-based axes and mechanisms inferred from other organs remain candidate modulators.

Testing a macrophage-mediated communication mechanism requires temporally restricted depletion, reconstitution or ligand-specific intervention, followed by quantitative analysis of the proposed receiver response and developmental output. Controls must exclude systemic inflammation, growth delay and nonspecific cell death.

Human fetal atlases are suited to defining immune-cell identity, timing and position, whereas mouse embryos and kidney explants can test selected functions [46,48]. Conventional kidney organoids lack stable embryonic immune populations and mature perfusion, limiting complete analysis of immune–vascular–nephron relationships. Coculture can test a specified local interaction only when immune-cell state, receiver identity and a quantitative developmental output are verified. Immune cells should therefore be treated as candidate modulators except for the restricted clearance and vascular effects supported by kidney-specific experiments.

### 5.7. Evidence-Graded Hypotheses and Distinguishing Predictions

A developmental communication model is useful when it distinguishes explanations that remain compatible with the observations. Co-expression, spatial proximity, pathway activation and morphological change represent different evidence classes. Table 2 evaluates specific mechanistic claims rather than assigning one status to an entire signaling pathway.

Claim category and evidence strength are evaluated separately. The categories defined in Section 3.2 describe whether a claim represents a technology-enabled observation, an experimentally supported mechanism, a literature reinterpretation or a prospective hypothesis. Prospective hypotheses may address a kidney-specific evidence gap, extend evidence from another developmental system or propose a cross-species comparison. These descriptors specify the question being tested without creating additional claim categories. Evidence level instead reflects the strength of support for the particular relationship stated in each row.

Evidence is graded for the claim stated in each row. Expression identifies candidate components, while spatial localization establishes anatomical plausibility. Functional support requires a perturbation that changes a receiver response or developmental outcome. Causal support requires cell-type- and stage-appropriate interventions linked to a defined morphogenetic effect, with rescue, epistasis or comparable evidence when feasible.

This distinction prevents strong pathway-level evidence from being transferred to a narrower, untested claim. GDNF–RET signaling is required for ureteric development, but the proposed composite sender domain remains untested by source-specific perturbation. WNT signaling is required for nephron induction, while a universal threshold or state-specific input–duration relation has not been established in vivo. Table 2 links each uncertainty to a prediction, an appropriate test and an outcome that would weaken the claim.

## 6. From Communication Inference to Testable Developmental Mechanisms

### 6.1. Sources of Error in Transcriptome-Based Communication Inference

Transcriptome-based communication analysis is sensitive to both expression measurement and cell classification. Widely expressed ligands and receptors generate many possible combinations, increasing the number of false-positive predictions. Low transcript abundance, limited sequencing depth and dropout can obscure genuine relationships. Communication scores are also affected by cell-population size, clustering resolution and expression thresholds. Dissociation-induced stress and immediate-early transcriptional responses may introduce signals that reflect tissue processing rather than development.

Ligand–receptor databases introduce a different set of constraints. Most are enriched for well-characterized pathways and extensively studied species, while multisubunit receptors, co-receptors, antagonists and ligand processing are represented less consistently [23]. Transcript abundance does not determine whether a ligand is translated, secreted, activated or retained within the ECM. Receptor mRNA likewise provides no direct evidence that the complete receptor complex is present or active. These limitations are particularly relevant to developmental tissues, where signaling range and response competence can change rapidly with cell state and anatomical position.

Spatial data reduce some ambiguity but create others. Mixed-cell spots and limited resolution can generate apparent co-localization, while genuine short-range relationships may be missed when rare cells or low-abundance transcripts are poorly detected. Cells occupying the same region may respond independently to a third signal rather than communicate directly with one another. Computational methods also differ in their treatment of expression specificity, receptor complexes and downstream targets. An interaction recovered by a single algorithm, dataset or clustering scheme should therefore remain a candidate. Reproducibility across reasonable analytical choices and independent, stage-matched datasets increases confidence but does not establish mechanism [7].

### 6.2. Tiered Validation of Communication Mechanisms in Kidney Development

Validation should begin with a defined morphogenetic question and specify the sender, receiver, developmental window and expected output. Without this connection, an expression relationship remains descriptive.

The levels in Table 2 guide validation of a specific claim rather than rank the importance of a pathway. Expression evidence gains value when stage-matched spatial localization confirms an anatomically plausible interaction. Spatial evidence is strengthened by receiver activation, while functional support requires cell-type- and stage-specific perturbation. Rescue or epistasis can then test specificity and causal order. The experiment should address the unresolved element of the claim, whether it concerns source identity, receiver competence, signaling range or developmental output. Additional evidence for the pathway as a whole cannot resolve a narrower uncertainty. For example, confirming that GDNF–RET signaling is required for branching does not establish that nephron progenitors and stroma form a functionally integrated sender domain. That question requires source-specific experiments that distinguish redundancy, temporal succession and spatial complementarity.

Anatomical and molecular plausibility requires stage-matched sender and receiver cells within an appropriate signaling range. Protein localization should confirm the ligand and complete receptor complex, while receptor activation or a specific downstream response should show that the receiver has responded.

**Figure 2:**
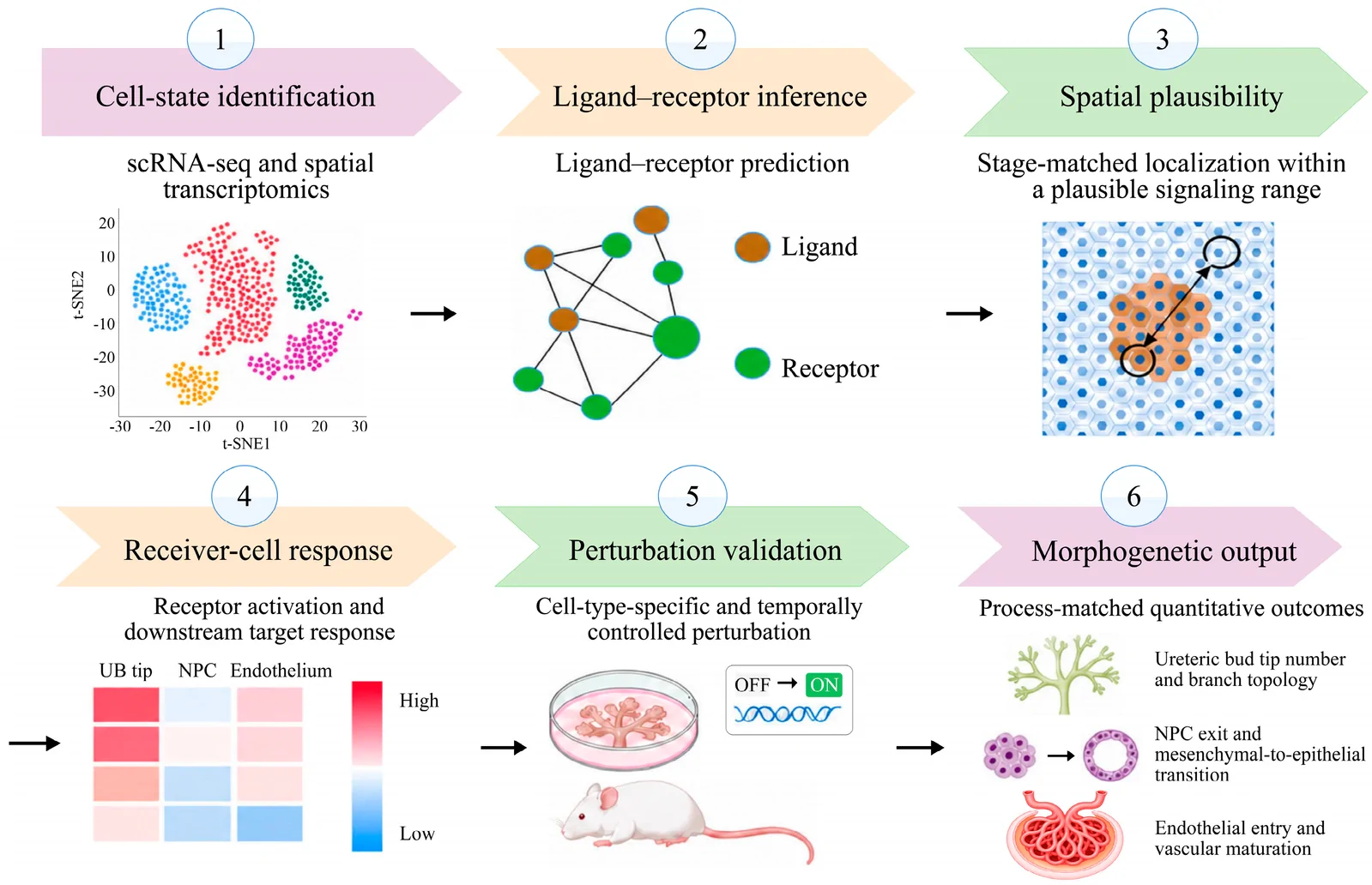
Evidence framework for evaluating predicted cell–cell communication during kidney development. This conceptual schematic does not propose a new general validation pipeline; it specifies how successive evidence levels should be linked to process-matched readouts in the developing kidney. Candidate interactions progress from cell-state assignment and ligand–receptor inference to stage-matched spatial localization, receiver activation, targeted perturbation and quantitative developmental outcomes. In the schematic t-SNE panel, colors denote illustrative cell-state clusters and do not represent a specific dataset.

Causal support requires cell-type-specific and, where feasible, temporally controlled perturbation of the ligand or receptor. Concordant changes in receiver state and a quantitative morphogenetic output strengthen the interpretation. Rescue and dose–response experiments help distinguish communication defects from altered viability or generalized growth inhibition. Outcomes should match the process under study, such as branch topology, progenitor exit or vascular maturation. This evidence sequence and its process-matched readouts are summarized in Figure 2.

### 6.3. Conservation, Divergence, and Model Boundaries in Human and Mouse Nephrogenesis

Mouse genetic studies provide most of the causal evidence for mammalian nephrogenesis. UB branching, nephron progenitor recruitment, mesenchymal-to-epithelial transition and nephron patterning have clear counterparts in humans, and the central involvement of GDNF–RET, WNT, FGF, BMP and Notch is broadly conserved [50]. This does not make the evidence interchangeable. Mouse lineage tracing and cell-type-specific perturbation can test origin, necessity and mechanism, whereas human fetal atlases primarily resolve cell identity, developmental timing and anatomical location.

Human fetal data should be interpreted at the level directly measured. Transcript detection establishes cellular presence, whereas spatial co-localization establishes anatomical plausibility. Neither trajectory analysis nor ligand–receptor inference demonstrates receptor activation or a causal developmental effect. Those conclusions require pathway readouts and controlled perturbation in an appropriate model.

Cross-species correspondence should be based on affected cell state, tissue position, morphological stage and direction of effect rather than on a shared marker or cluster name. A mouse mechanism can guide interpretation of human fetal localization, but the localization does not validate the causal chain in humans. Organoids and explants can test selected responses under defined conditions; their findings remain model-specific unless the relevant cell state and tissue organization match fetal kidney. Table 3 summarizes these evidential boundaries and their implications for CAKUT.

### 6.4. Organoids as Question-Specific Models

Human pluripotent-stem-cell-derived kidney organoids provide a manipulable system for studying early lineage induction, nephron segmentation and human genetic variants. They should not, however, be treated as complete fetal kidneys. Most protocols generate nephron-like structures with variable stromal and endothelial populations, while UB branching, stable perfusion, immune-cell entry and urine drainage remain incomplete [9,55,56].

Cell identity is a central constraint. Organoid cells may share markers with fetal kidney populations while retaining culture-induced stress, abnormal cell-cycle states or intermediate transcriptional features [51,52]. Off-target neural-like and myogenic populations may also arise. Annotation should therefore combine transcriptomic, protein, spatial and functional evidence, using stage-matched human fetal tissue where possible [8,31].

Organoids also differ from fetal kidneys in perfusion, matrix composition, geometry and developmental timing. The absence of mature perfusion affects oxygen availability, nutrient supply, shear stress and waste clearance [55]. Matrix conditions, cell line, induction batch and WNT exposure influence cell composition and nephron formation, so days in culture cannot be equated directly with gestational age [9,57].

Organoids are suited to testing receiver competence, variant effects and defined dose–time responses [58]. Questions involving branching geometry, perfusion, immune recruitment or drainage require complementary models [59,60]. A response to an exogenous ligand demonstrates competence under the culture condition, not the operation of the same communication axis in human development.

### 6.5. Analytical Discrepancies, Model-Dependent Differences and Unresolved Biological Questions

Kidney-development studies differ for several reasons, and not every difference represents a biological conflict. Dataset composition can change with developmental stage, sampled region, dissociation method, sequencing platform, clustering resolution and cell-annotation rules. Comparisons should therefore begin by asking whether the studies examined equivalent cell states, anatomical regions and developmental intervals. A difference that disappears after such alignment is better regarded as an analytical discrepancy than as evidence of distinct developmental mechanisms.

Human fetal kidney studies illustrate this problem. Menon et al. recovered broad developmental trajectories, whereas Hochane et al. resolved several transcriptionally distinct NPC states [5]. Spatial reconstruction subsequently placed progenitor and early nephron states along a continuous anatomical sequence [25]. These observations are not mutually exclusive: clustering can identify reproducible intervals within a continuous transition. The available spatial evidence favors a continuum containing distinguishable states over stable progenitor sublineages, but it does not provide clonal evidence. Stage-matched profiling with a shared analytical pipeline, combined with spatially resolved lineage or temporal fate measurements, would be required to distinguish stable lineage restriction from reversible state variation.

Some differences arise because experimental models interrogate different variables. Mouse nephron progenitor cultures treated with low or high CHIR99021 reveal dose-dependent responses to downstream β-catenin activation [26]. Human fetal maps identify progenitor states in defined anatomical positions but do not measure extracellular WNT9B concentration or establish a causal response threshold. These studies therefore do not support opposing mechanisms. Together, they favor a model in which WNT response depends on input strength, exposure duration and receiver state rather than a fixed threshold shared by all progenitors. A direct test would expose stage-matched, state-defined progenitors to a ligand-dose-by-duration matrix, distinguish receptor-mediated stimulation from downstream pathway activation and quantify early β-catenin activity before commitment, proliferation or survival diverges.

The cellular source of GDNF remains an unresolved biological question. Classical genetic studies established the requirement for mesenchymal GDNF–RET signaling during UB induction and branching [49]. Later transcriptomic and spatial analyses detected Gdnf in both nephron progenitors and nephrogenic-zone stroma [11]. These findings revise the anatomical definition of the candidate sender domain but do not establish the functional contribution of each source. A composite progenitor–stromal domain is currently better supported at the expression level, whereas lineage-specific necessity remains undetermined. Temporally controlled Gdnf deletion and source-specific rescue in Six2- and Foxd1-lineage cells on the same genetic background would distinguish redundant, sequential and stage-restricted functions.

Reported human–mouse differences require similar calibration. Human fetal studies cover a broader developmental interval, whereas mouse studies often examine narrower stages and permit lineage-restricted perturbation. Differences in stromal or progenitor states may therefore reflect stage alignment, anatomical sampling or annotation rather than species divergence. A species-specific interpretation is justified only when matched cell states occupy comparable anatomical positions, show a consistent difference under a joint analytical pipeline and produce a corresponding functional difference in an appropriate model.

Mechanical regulation represents an evidence gap rather than a conflict between equivalent studies. Mouse genetic studies implicate Hippo–YAP/TAZ and β1-integrin in UB development, whereas cell, three-dimensional culture and kidney-explant experiments support a role for FAK–Src signaling in collecting-duct morphogenesis [39,40,41]. These findings do not establish that matrix stiffness modifies RET responsiveness at a fixed GDNF input. Time-resolved measurements of tissue mechanics, RET activity and branch behavior are needed to determine whether mechanical changes alter signaling competence or arise as a consequence of branch growth.

### 6.6. Developmental Communication and Receiver Competence in CAKUT: An Evidence Hierarchy

Variants associated with CAKUT may directly disrupt ligand production, receptor function or pathway activation, or indirectly alter communication by changing cell identity and receiver competence. Patient association, human fetal localization, functional assays and mouse genetics represent distinct evidence levels. RET is a direct signal-reception component, whereas EYA1, SIX1, PAX2 and HNF1B should be classified primarily as competence regulators unless a specific sender–receiver defect has been demonstrated [53,54,61].

#### 6.6.1. RET: Defective Signal Reception in the UB

Among the genes considered here, RET provides the clearest link between a CAKUT-associated gene and signal reception. Human RET variants have been associated with renal agenesis, but their penetrance and functional effects vary; one fetal series also demonstrated altered RET phosphorylation for selected variants [62]. Mouse Ret loss establishes a causal requirement in UB induction and early branching [49]. Human fetal localization identifies the relevant receiver compartment and stage, but cannot determine the effect of an untested patient variant. The human association and the mouse GDNF–RET mechanism are therefore related evidence classes rather than a single causal chain.

#### 6.6.2. EYA1–SIX1: Impaired Developmental Competence of the Metanephric Mesenchyme

The transcriptional regulators EYA1 and SIX1 form a complex rather than a ligand–receptor pair. Human EYA1 and SIX1 variants are associated with branchio-oto-renal syndrome and show variable renal phenotypes; selected SIX1 variants disrupt EYA1–SIX1 or SIX1–DNA interactions in vitro [63,64]. Eya1 loss in mice prevents normal kidney induction and provides causal developmental evidence [65]. These findings support impaired establishment and competence of the metanephric mesenchyme. Effects on GDNF production and epithelial–mesenchymal communication are downstream consequences inferred mainly from animal models, not a direct human communication defect.

#### 6.6.3. PAX2, HNF1B, and WNT4: Defects in Cell Identity and Differentiation Programs

The genes considered in this section occupy different positions in the evidence hierarchy. PAX2 variants are associated with renal hypoplasia and show marked phenotypic variability; combined human and mouse analysis links reduced PAX2 function to developmental cell survival [66]. HNF1B variants are associated with a broad spectrum of cystic and dysplastic phenotypes, while functional studies support variant-dependent effects on transcriptional and epithelial differentiation programs [67]. Neither factor constitutes a ligand–receptor axis. WNT4 acts more directly in nephron epithelialization: mouse loss establishes developmental necessity [13], whereas human evidence is largely restricted to rare syndromic loss-of-function variants [68]. These data should not be generalized to most isolated CAKUT.

#### 6.6.4. SLIT2–ROBO2: Loss of Spatial Control over UB Induction

Signaling through the SLIT2–ROBO2 axis links spatial control to UB induction. *Slit2* or *Robo2* loss in mice expands the *Gdnf* domain and permits supernumerary buds, establishing a causal mechanism in that model [69]. Human ROBO2 disruption and rare ROBO2 or SLIT2 variants have been associated with vesicoureteral reflux and other CAKUT phenotypes, but segregation, penetrance and functional support vary among reports [70,71]. Human fetal localization can identify plausible source and receiver domains; it does not show that a patient variant alters GDNF distribution. Application of the mouse mechanism to human CAKUT therefore remains a cross-species mechanistic inference. The mechanistic chains and evidential boundaries for the CAKUT-associated genes discussed here are summarized in Appendix.

## 7. Summary and Outlook

Single-cell and spatial studies have refined the cellular and anatomical assignment of established kidney-development signals. Their clearest contributions include a broader candidate GDNF sender domain, state- and exposure-dependent interpretation of WNT responses, and a kidney-specific role for stromal NTN1 in vascular organization. These findings are supported at different evidential levels and should not be treated as equivalent demonstrations of niche-level causality.

The dynamic developmental niche framework provides a consistent approach for evaluating local communication by separating pathway requirement from sender identity, receiver competence, developmental timing, anatomical range and morphogenetic response. It allows established mechanisms, spatially plausible relationships and unresolved hypotheses to be compared within the same evidence structure. Although the framework remains pre-quantitative, it identifies the variables and causal gaps that future studies must address.

Progress will require experiments directed at the unresolved component of each claim, including source-specific perturbation, state-aligned input–response analysis and time-resolved measurement of signaling and morphogenesis. For CAKUT-associated variants, the framework can distinguish defects in signal production, receptor activation, receiver competence, spatial organization and downstream morphogenesis. This distinction can help prioritize functional studies and select appropriate organoid, explant or animal models. In kidney organoids, developmental fidelity should be evaluated through sender and receiver states, spatial relationships, pathway activation and process-matched morphogenetic outputs rather than lineage markers alone. These applications support mechanistic interpretation and model evaluation, but they do not yet predict clinical outcomes or direct patient treatment.

## Figures and Tables

**Table 1:** Evidence map for major communication axes in embryonic kidney morphogenesis.

Process and Stage	Sender → Receiver	Signal–Receptor Axis	Established Developmental Output	Evidence Boundary and References
UB induction and branching; mouse, from E10.5	NPC and stromal compartments → RET+ UB tip	GDNF → RET–GFRα1	Bud outgrowth and branching	Six2- and Foxd1-lineage contributions remain unresolved [11].
Ureteric branch-pattern control; mouse, E11.5–E18.5	Mesenchymal/stromal compartments → ALK3 + UB epithelium	BMP → ALK3	Branch pattern and pSMAD1 response	Ligand source, range and stage remain unresolved [12].
Nephron induction; mouse, from approximately E11.5	UB epithelium → cap mesenchyme/NPCs	WNT9B → Frizzled–LRP5/6–β-catenin	Wnt4 induction, pretubular aggregates and NPC commitment	In vivo input intensity and exposure duration are undefined [2].
Nephron epithelialization; pretubular aggregate to renal vesicle	Induced NPCs → induced NPCs/pretubular aggregates	WNT4 → Frizzled–β-catenin-associated signaling	MET and renal-vesicle formation	Receptor composition and autocrine versus short-range action remain unresolved [13].
Early nephron differentiation and segmentation	Adjacent nephron epithelium → nascent nephron epithelium	JAG1/DLL1 → NOTCH1/2	Early differentiation and segment-fate selection	Ligand-specific sender assignment and human causality remain incomplete [14].
Stromal regulation of nephrogenesis; mouse, E13.5–E18.5	Distal ureteric/collecting epithelium → FOXD1-lineage stroma	SHH → PTCH1–SMO–GLI	Stromal organization, NPC distribution and nephron number	Dominant SHH source and complete source-to-output chain remain partly resolved [15].
Stromal inhibition in altered signaling contexts; mouse and organoid models	FOXD1-lineage stroma → unresolved receiver	CXCL12/WNT5A → candidate CXCR4/Frizzled responses	Stromal expansion and reduced nephron formation	Evidence is context-dependent and does not establish a normal physiological axis [16].
Glomerular endothelial recruitment; late embryonic/perinatal mouse	Podocyte lineage → KDR/VEGFR2+ endothelium	VEGFA → KDR/VEGFR2	Endothelial recruitment, survival and filtration-barrier development	Findings should not be generalized to all renal vascular entry [17].
Mesangial recruitment; S-shaped body to glomerular tuft	Glomerular endothelium → PDGFRβ+ mesangial progenitors	PDGF-B → PDGFRβ	Mesangial recruitment and capillary-tuft organization	Later state-specific receiver responses remain less defined [18].
Renal arterial patterning and maturation; embryonic mouse	FOXD1-lineage stroma → UNC5B+ renal endothelium	NTN1 → UNC5B	Renal arterial branching, mural-cell coverage and vascular maturation	Vascular causality is supported, but the causal relationship with nephron abnormalities remains unresolved [19].

Abbreviations: ALK3, activin receptor-like kinase 3 (BMPR1A); BMP, bone morphogenetic protein; DLL1, Delta-like ligand 1; GDNF, glial cell line-derived neurotrophic factor; GFRα1, GDNF family receptor alpha 1; JAG1, Jagged canonical Notch ligand 1; KDR, kinase insert domain receptor; LRP5/6, low-density lipoprotein receptor-related proteins 5 and 6; MET, mesenchymal-to-epithelial transition; NPC, nephron progenitor cell; PDGF, platelet-derived growth factor; PDGFRβ, platelet-derived growth factor receptor beta; pSMAD1, phosphorylated SMAD1; SHH, sonic hedgehog; UB, ureteric bud; VEGFA, vascular endothelial growth factor A; NTN1, netrin 1; RET, RET proto-oncogene receptor tyrosine kinase; UNC5B, unc-5 netrin receptor B.

**Table 2:** Claim classification, evidence levels and distinguishing tests for developmental communication models.

Claim, Category and Evidence Level	Distinguishing Prediction	Critical Test and Readouts	Decision Rule	Evidence Basis
ECM mechanics may regulate RET responsiveness; prospective hypothesis (kidney-specific evidence gap), Level 1	Mechanical loading alters RET activity before branch geometry changes	Manipulate matrix mechanics; measure early RET activity and growth-normalized branching	Supports: RET changes precede branching. Weakens: Growth, viability or ligand availability explains the phenotype.	[39,40,41]
NPC state may modify the WNT input–response relation; prospective hypothesis (kidney-specific evidence gap), Level 1 for state dependence	State-aligned NPCs retain different responses to WNT intensity or duration	Apply a ligand-dose-by-duration matrix; measure early β-catenin activity and commitment	Supports: State-specific responses persist after control. Weakens: Aligned states respond equivalently.	[26]
NPCs and nephrogenic-zone stroma may form a composite GDNF source; literature reinterpretation, Level 3	Six2- and Foxd1-lineage sources make distinct or complementary contributions	Perform lineage- and time-controlled deletion with source-specific rescue	Supports: Source-specific defects are rescued. Weakens: No reproducible source effect remains after alignment.	[11,49]
Stromal boundaries may regulate ureteric signaling through tissue geometry; prospective hypothesis (kidney-specific evidence gap), Level 1	Boundary disruption precedes altered GDNF distribution, RET activity and branch position	Perturb a defined stromal/ECM component; track geometry, signaling and branching	Supports: Geometric change occurs first and restoration rescues patterning. Weakens: Signaling or growth changes precede geometry.	[37,38]
Macrophages contribute to the clearance of misplaced or dying NPCs; experimentally supported mechanism, Level 4 within the tested mouse model	A clearance defect precedes nephrogenic-zone disorganization	Use stage-restricted depletion/reconstitution; measure uptake, misplaced/apoptotic NPCs and niche organization	Supports: Clearance and organization recover after reconstitution. Weakens: Inflammation, growth delay or nonspecific death explains the phenotype.	[48]
Macrophages contribute to renal vascular connectivity; experimentally supported mechanism, Level 4 within the tested kidney-explant model	Connectivity declines before generalized tissue deterioration	Use stage-restricted depletion/reconstitution; measure endothelial continuity and branch points	Supports: Connectivity declines early and recovers. Weakens: The phenotype follows inflammation, cell death or reduced growth.	
Human and mouse kidneys may use different cellular sources for a conserved function; prospective hypothesis (comparative), Level 2–3	A source or timing difference persists after alignment by state, position and morphology	Jointly map stage-matched sources; compare perturbation, receptor activation and morphology	Supports: The difference persists after alignment with concordant receiver responses. Weakens: The difference disappears or rests only on transcript detection.	[50]

Abbreviations: ECM, extracellular matrix; GDNF, glial cell line-derived neurotrophic factor; NPC, nephron progenitor cell; RET, RET proto-oncogene receptor tyrosine kinase; WNT, WNT-family signaling. Note: Claim category describes the origin and current status of an interpretation, whereas evidence level reflects the strength of support for the specific relationship stated in each row. Parenthetical descriptors identify the question addressed by a prospective hypothesis and do not represent additional claim categories. Evidence levels should not be transferred from an established pathway to a narrower, untested mechanism. Evidence levels: Level 1, direct kidney evidence is absent or insufficient; Level 2, candidate components are expressed in defined populations; Level 3, sender and receiver are localized within a plausible developmental window and range; Level 4, perturbation changes a receiver response or developmental outcome, but specificity or rescue remains incomplete; Level 5, cell-type- and stage-appropriate causal evidence links communication to a defined morphogenetic outcome.

**Table 3:** Systematic comparison of human and mouse kidney development and the limits of cross-model interpretation.

Dimension	Mouse	Human Fetal Kidney	Interpretive Boundary	References
Developmental timing	Nephrogenesis is embryonic and ends shortly after birth	Nephrogenesis continues into late gestation	Align by morphology, anatomical position and cell state rather than proportional age	[50]
Nephron progenitor states	Lineage dependencies and causal transitions can be tested genetically	Continuous states are resolved, but lineage stability and causal transitions remain uncertain	State maps do not establish functional WNT responses	
Stromal populations	Foxd1-lineage origin and function can be tested	Multiple spatial states are resolved, but lineage correspondence remains incomplete	Align populations by position, transcriptional state and function	[28,29]
Pathway function and cellular source	Cell-specific genetics can establish source and receiver function	Source assignments rely mainly on transcript detection and spatial association	Shared expression does not establish conserved source, activation or function	[23,33]
Vascular maturation	Endothelial entry, arterialization and mural-cell recruitment can be followed experimentally	A longer developmental interval contains more diverse endothelial and mural states	Position and expression do not establish receptor activation or causal nephron effects	[42,43]
Immune cells	Origins can be lineage-traced, but kidney-specific functional evidence remains limited	Atlases define immune-cell presence, timing and distribution	Developmental function requires perturbation and a receiver-level response	[46,47]
Kidney organoids	Findings can be compared with causal mouse models	Human cellular states and variant effects can be modeled, but tissue organization remains incomplete	Benchmark against stage-matched human fetal kidney	[51,52]
CAKUT interpretation	Genetic perturbation can be linked to morphogenesis	Patient genetics supports association; fetal tissue provides localization	Report association, localization, model perturbation and human developmental causality separately	[53,54]

Abbreviations: CAKUT, congenital anomalies of the kidney and urinary tract; WNT, WNT-family signaling.

## Data Availability

This review is based on published literature and publicly available information. No new datasets were generated or analyzed for this article.

## Abbreviations

The following abbreviations are used in this manuscript:

ANGPT	angiopoietin
BMP	bone morphogenetic protein
CAKUT	congenital anomalies of the kidney and urinary tract
ECM	extracellular matrix
FAK	focal adhesion kinase
FGF	fibroblast growth factor
GDNF	glial cell line-derived neurotrophic factor
GFRα1	GDNF family receptor alpha 1
KDR	kinase insert domain receptor
LRP5/6	low-density lipoprotein receptor-related proteins 5 and 6
MET	mesenchymal-to-epithelial transition
NPC	nephron progenitor cell
NTN1	netrin 1
PDGF	platelet-derived growth factor
PDGFRβ	platelet-derived growth factor receptor beta
PTCH1	patched 1
RET	RET proto-oncogene receptor tyrosine kinase
scRNA-seq	single-cell RNA sequencing
SHH	sonic hedgehog
SMO	smoothened
TCF	T-cell factor
TEK	TEK receptor tyrosine kinase
UB	ureteric bud
UNC5B	unc-5 netrin receptor B
VEGF	vascular endothelial growth factor
VEGFA	vascular endothelial growth factor A
VEGFR2	vascular endothelial growth factor receptor 2
WNT	WNT-family signaling
YAP/TAZ	yes-associated protein and transcriptional coactivator with PDZ-binding motif

## Appendix A

**Table A1:** Mechanistic chains and evidential boundaries for CAKUT-associated genes.

Gene or Pathway	Cellular Site and Developmental Window	Developmental and Clinical Phenotype	Causal Evidence	Evidential Boundary and References
*RET*	Ureteric epithelium; bud induction and early branching	Renal agenesis and collecting-system abnormalities with variable penetrance	Variant-specific phosphorylation assays; strong causal evidence from Ret-deficient mice	Fetal localization identifies the receiver compartment but not the effect of an individual variant [62].
*EYA1–SIX1*	Metanephric mesenchyme; kidney induction	Renal agenesis, hypoplasia, dysplasia and collecting-system abnormalities in BOR syndrome	Selected SIX1 variants impair molecular interactions; Eya1 loss is causal in mouse	This is primarily a competence defect; communication effects are downstream cross-species inferences [63,64,65].
*PAX2*	Progenitor identity, competence and survival; induction through nephron formation	Hypoplasia, dysplasia and variable cystic abnormalities with incomplete penetrance	Human–mouse studies support altered survival; mouse models provide causal developmental evidence	PAX2 is not a direct communication axis, and expression does not validate variant function [66].
*HNF1B*	Differentiating nephron and collecting-system epithelia	Cystic kidneys, dysplasia and abnormal tubules with broad genotype–phenotype variability	Variant assays and developmental models support allele-dependent functional effects	A specific intercellular communication mechanism has not been established [67].
*WNT4*	Induced nephron progenitors; pretubular aggregate and renal-vesicle formation	Impaired epithelialization and renal dysgenesis in rare syndromic loss of function	Human variant functional support; strong causal evidence from Wnt4-deficient mice	Rare syndromic evidence should not be generalized to most isolated CAKUT [68].
*SLIT2–ROBO2*	Spatial restriction of the Gdnf and bud-induction domains	Supernumerary buds, reflux and duplicated collecting systems; variable human penetrance	Slit2/Robo2 loss is causal in mouse; functional support for human variants is uneven	Altered GDNF distribution is established in mouse, not affected human fetal kidneys [69,70,71].

BOR, branchio-oto-renal syndrome.
